# Transfer learning for photonic delay-based reservoir computing to compensate parameter drift

**DOI:** 10.1515/nanoph-2022-0399

**Published:** 2022-10-18

**Authors:** Ian Bauwens, Krishan Harkhoe, Peter Bienstman, Guy Verschaffelt, Guy Van der Sande

**Affiliations:** Applied Physics Research Group, Vrije Universiteit Brussel, Pleinlaan 2, 1050 Brussels, Belgium; Photonics Research Group, Department of Information Technology, Ghent University-IMEC, Technologiepark Zwijnaarde 126, 9052 Ghent, Belgium

**Keywords:** feedback, optical injection, photonic reservoir computing, semiconductor lasers, transfer learning

## Abstract

Photonic reservoir computing has been demonstrated to be able to solve various complex problems. Although training a reservoir computing system is much simpler compared to other neural network approaches, it still requires considerable amounts of resources which becomes an issue when retraining is required. Transfer learning is a technique that allows us to re-use information between tasks, thereby reducing the cost of retraining. We propose transfer learning as a viable technique to compensate for the unavoidable parameter drift in experimental setups. Solving this parameter drift usually requires retraining the system, which is very time and energy consuming. Based on numerical studies on a delay-based reservoir computing system with semiconductor lasers, we investigate the use of transfer learning to mitigate these parameter fluctuations. Additionally, we demonstrate that transfer learning applied to two slightly different tasks allows us to reduce the amount of input samples required for training of the second task, thus reducing the amount of retraining.

## 1 Introduction

With the tremendously fast growth of the amount of information in our digital age, we are becoming ever increasingly reliable on machine learning to analyze this large quantity of information [1, 2]. Currently, this is typically performed on digital hardware. However, due to the breakdown of Moore’s law [3], there is considerable interest to resort to analog systems to perform the computations required for machine learning. One example of such a machine learning strategy, suited for analog machines, is reservoir computing (RC). Reservoir computing systems were originally based on recurrent neural networks and consist of a large amount of nodes with random but fixed interconnections. An RC system is generally divided in three separate layers: an input layer, a reservoir layer and an output layer. The input layer is used to inject the data into the reservoir. In the reservoir layer, the data will be processed in a complex, non-linear dynamical system and sent through to the output layer. In this output layer, linear weights are used to calculate the reservoir’s output. These weights are optimized during the training phase. Note that the internal weights of the reservoir itself are not being optimized and remain constant during training. This results in RC systems being very simple to train and makes them very time and energy efficient. RC systems have so far been successfully applied to several tasks, including non-linear channel equalization [4], time-series predictions [5–7] and speech recognition [8–10]. In this work, we implement a reservoir computing system by using opto-electronic components. These opto-electronic systems offer several advantages, including fast information processing rates combined with low energy consumption [11, 12]. However, a problem with the physical implementations of photonic reservoir computing systems is their parameter drift during operation. For example, varying room temperatures lead to temperature-induced internal length differences. This will lead to a difference in the feedback phase and ultimately results in a loss of performance, which we want to avoid as much as possible. Several publications have investigated the effects of these influences, including possible techniques to counteract this. This can for example be done by actively adapting the length changes of a coherent linear Fabry–Perot resonator using a control loop [13], reducing the noise sensitivity of the RC performance by improving the pre-processing of data in the input layer [14] or by expanding the output layer to improve performance [15]. However, further improvements are possible, which is why there is considerable interest in developing other approaches. In this paper, we numerically explore the use of a novel learning paradigm introduced in [16], referred to as *transfer learning*, applied to photonic reservoir computing. This technique builds upon the conventional training method and tries to enhance it by reusing information gained from previous training procedures and applying this information when training on different, but still similar problems. It offers the advantage of being able to find weights with a minimal amount of required retraining for the new problem. In this numerical study, we apply this transfer learning technique to photonic delay-based RC systems with semiconductor lasers.

This paper is organised as follows. Section 2 gives a short introduction on delay-based reservoir computing and discusses the numerical model that we use to implement this RC system. In Section 3, we introduce and discuss two training methods: conventional training and transfer learning. In Section 4, we compare the RC performance when using transfer learning and conventional training. In Section 5, we investigate whether transfer learning can be used to mitigate influences of small changes in the feedback phase of the RC system on the performance of the RC itself. In Section 6, we investigate the use of transfer learning when multiple parameters are varied, namely the injection rate, feedback rate and excess pump current. Section 7 gives a conclusion of the previous results of the paper.

## 2 Numerical implementation of delay-based reservoir computing using semiconductor lasers

**Figure 1:**
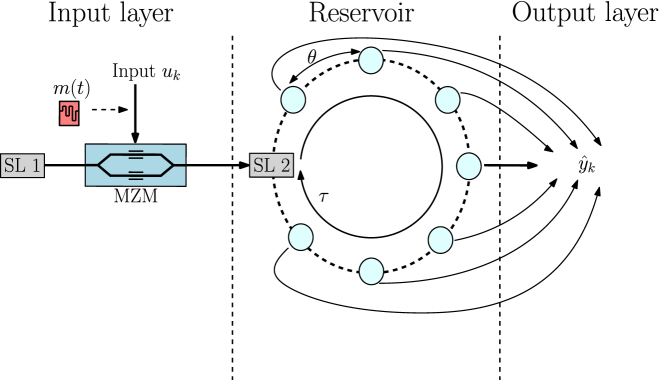
Illustration of a delay-based RC system using a semiconductor laser (SL). SL 1 drives the system and SL 2 is used to simulate the reservoir. A mask *m*(*t*) is used to encode the input data sample *u*_*k*_, which is optically injected into the reservoir using a Mach–Zehnder modulator (MZM). Also shown here is the node separation *θ* and delay time *τ*. The virtual nodes are represented by the light blue circles and the predicted target data by ${\hat{y}}_{k}$ .

In this paper, we use a reservoir computing system based on semiconductor lasers (SL) with delayed feedback [17]. Various types of photonic or electronic reservoir computing systems have already been realized using this delay-based technique [17–20]. In Figure 1, we show the structure of our RC system. It is composed of three layers: the input layer, the reservoir layer and the output layer. The input layer consists of a semiconductor laser coupled with an unbalanced Mach–Zehnder modulator which optically injects input samples into the reservoir layer. Before the data is injected into the reservoir, the input samples are first encoded via a mask *m*(*t*). The reservoir layer consists of a single-mode semiconductor laser (SL) with delayed optical feedback with a delay length *τ*. The time trace of the light intensity emitted by the SL is measured by a photodetector after the reservoir layer. This time trace is then sampled and used in the output layer, where the weights are calculated. This procedure is explained in further detail in Sections 3.1 and 3.2 [11, 21].

The numerical simulations for our delay-based RC system are based on the following rate-equations [22]

(1) $$\begin{array}{ll} \frac{dE\left(t\right)}{dt}=\frac{1}{2}\left(1+i\alpha \right)\xi N\left(t\right)E\left(t\right)+\eta E\left(t-\tau \right){\text{e}}^{-\text{i}\phi_{FB}}\\ & \;+{\tilde{F}}_{\beta }\left(t\right)+\mu E_{\mathrm{inj}}\left(t\right) \end{array}$$

(2) $$\frac{dN\left(t\right)}{dt}=\frac{\Delta I}{e}-\frac{N\left(t\right)}{\tau_{c}}-\left[g+\xi N\left(t\right)\right]{\left|E\left(t\right)\right|}^{2},$$

with *E*(*t*) the complex valued slowly-varying amplitude of the electric field of the laser and *N*(*t*) the excess amount of available carriers, both of which are dimensionless parameters. *ξ* and *g* represent the differential gain and threshold gain of the laser. *α* is the linewidth enhancement factor. *η* and *μ* are the feedback rate and the injection rate parameters. Δ*I*/*e* is the excess pump current rate normalized with the elementary charge, with Δ*I* = *I* − *I*_thr_ and where *I* is the injected pump current and *I*_thr_ the threshold pump current. *ϕ*_*FB*_ is the feedback phase. Complex Gaussian white noise is added to the system by ${\tilde{F}}_{\beta }\left(t\right)$ to model spontaneous emission noise, with $\left\langle {\tilde{F}}_{\beta }\left(t\right)\right\rangle =0$ and $\left\langle {\tilde{F}}_{\beta }\left(t\right){\tilde{F}}_{\beta }{\left(t^{\prime }\right)}^{*}\right\rangle =\beta /\tau_{c}\;\delta \left(t-t^{\prime }\right)$ . In this term, the spontaneous emission noise strength is controlled by *β* and the carrier lifetime is represented by *τ*_*c*_. The data injection is performed optically by *E*_inj_(*t*), where its slowly varying envelope is given by $E_{\mathrm{inj}}\left(t\right)=\epsilon \left(1+{\text{e}}^{\text{i}B\left(t\right)}\right)$ . This injection occurs on the same frequency as the free running laser so that the injection frequency detuning is zero and remains constant in this work [23]. *ϵ* is the amplitude of the injected electric field, and *B*(*t*) represents the injected data signal,

(3) B(t)=AS(t)+Φ,

with *A* and Φ the modulation amplitude and bias, originating from the Mach–Zehnder modulator. *S*(*t*) is defined as

(4) $$S\left(t\right)=m\left(t\right)*\underset{k}{\sum }u_{k}\delta \left(t-k\tau \right),$$

with * the convolution operator, *u*_*k*_ the *k-*th input sample from a total of *n* input samples, *δ* the Dirac delta function and *m*(*t*) the mask. Because we use delay-based reservoir computing with delayed optical feedback, we first have to time-multiplex the input data. This is performed by using the mask *m*(*t*), so that every input sample *u*_*k*_ is injected into the reservoir for a given time length. The mask consists of a piecewise constant function, with sublevels randomly selected from 5 values: $\left[0,0.25,0.5,0.75,1\right]$ . These sublevels are kept piecewise constant with a duration equal to the node separation, *θ*, so that the total duration of this mask is equal to the number of virtual nodes, *N*, multiplied with the node separation *θ*. Every input data sample is injected for duration equal to the mask length, *Nθ*, during which this input data sample is multiplied with the mask, resulting in a masked input signal. The node separation *θ* and delay length *τ* are also held constant in all simulations. We use here values *θ* = 20 ps and *τ* = 4 ns that have proven to work well for our laser based RC system [24]. These parameter values lead to the number of virtual nodes being equal to 200. In this paper, we have opted to choose the period of the mask equal to the delay length *τ*, so that *τ* = *Nθ*. This is done purely out of simplicity, even though an improved performance can be found when we introduce a small mismatch in the mask length [25]. A summary of all used parameters can be found in Table 1, which are taken from [23].

**Table 1:** Parameters, together with their respective values, used in the simulations. Parameters marked with * can be different from given values, when stated.

Parameter	Symbol	Standard value
Amount of virtual nodes	*N*	200
Node separation	*θ*	20 ps
Linewidth enhancement factor	*α*	3
Threshold gain	*g*	1 ps^−1^
Differential gain	*ξ*	5 × 10^−9^ ps^−1^
Spontaneous emission noise factor	*β*	$\approx 10^{2}$
Carrier lifetime	*τ*_*c*_	1 ns
Threshold pump current	*I*_thr_	16 mA
Excess pump current rate*	$\frac{\Delta I}{e}$	1.02 × 10^5^ ps^−1^
Feedback rate*	*η*	7.8 ns^−1^
Injection rate*	*μ*	98.1 ns^−1^
Amplitude of injected field	*ϵ*	100
Feedback phase*	*ϕ*_*FB*_	0
Modulation amplitude of MZM	*A*	$\frac{\pi }{2}$
Bias voltage of MZM	Φ	$\frac{\pi }{4}$

## 3 Training procedures

In this section, we explain the two different training procedures we use in this paper: conventional training and transfer learning.

### 3.1 Conventional training procedure

We obtain the output weights **w** corresponding to the *N* nodes of the reservoir in the training phase, where the training is performed off-line. To this aim, we use the normalized state matrix, **A**, of the RC system and the expected data, **y**. Because the input samples are time-multiplexed in the input layer, we have to de-multiplex the output, represented by the light intensity |*E*(*t*)|^2^ measured by a photodetector at the output of SL 2. This de-multiplexing is performed by sampling the intensity time trace at every *θ* time interval for each input data sample. The *N* sampled intensities are stored in the columns of **A**, which is done for all the input samples, stored in the rows of **A**. The resulting matrix is referred to as the state matrix **A** with dimensions (*n* × (*N* + 1)), where *n* and *N* represent the number of input samples and the number of nodes of the RC system. In this matrix an additional bias node has been added in order to account for a possible offset in the data. The state matrix can be used to find the weights **w** for the *N* nodes of the reservoir and the additional bias node, to match with the expected data samples **y**. This is performed using a least squares minimization and results in predicted values for the data samples $\hat{y}$ , which we want to make as close to **y** as possible. In matrix notation, this translates to

(5) $$\hat{y}=Aw.$$

These weights dictate the scaling of the individual nodes of the RC network for the state matrix **A**. In practice, the weights **w** can be calculated by minimizing the squared error between the predicted value for the data samples $\hat{y}$ , resulting from the matrix multiplication in Eq. (5), and the expected data samples **y**. The practical implementation of this can performed by calculating the real Moore–Penrose pseudoinverse (denoted by the symbol †) for Eq. (5):

(6) $$w=A^{†}y.$$

This previous equation can be simplified even further by making use of the fact that the state matrix contains the light intensity and is thus real-valued, so that

(7) $$w={\left(A^{T}A\right)}^{-1}A^{T}y.$$

Once the weights have been calculated in the training phase, we test how the RC performs on unseen data, which is referred to as the test phase. In order to quantify this performance, we use the normalized mean squared error (NMSE) between the expected output **y** and predicted output $\hat{y}$ .

(8) $$NMSE\left(\hat{y},y\right)=\frac{\left\langle {\left(y-\hat{y}\right)}^{2}\right\rangle }{\left\langle {\left(y-\left\langle y\right\rangle \right)}^{2}\right\rangle }.$$

### 3.2 Transfer learning procedure

The concept of transfer learning builds upon the conventional training of weights, explained in Section 3.1. The main advantage transfer learning offers is that when we have already performed training on a particular task, we can reuse this information for different, but still similar tasks. Therefore, a key difference is that instead of one general training dataset, we now have two different training datasets, referred to as the training source and training target dataset. The training source dataset $D_{S}$ contains the information of the first task, of which we assume here to have a lot of information, i.e. many data samples. The training target dataset $D_{T}$ contains information of the second task, which is similar to the first task, and typically contains fewer data samples than the training source dataset. This could be due to an inherently limited amount of data on the task or because we want to minimize the required amount of training due to energy or time constraints.

We follow [26] in order to implement the transfer learning, which can be summarized as follows. One starts by finding the weights corresponding to the training source dataset, as explained in Section 3.1 and which results in the training source weights **w**_*S*_. The weights for the training target dataset are expected to be similar to those of the training source dataset, because both tasks are similar, and therefore will only require a small correction. The training target weights are therefore defined as

(9) $$w_{T}=w_{S}+\delta w,$$

such that the expected target data **y**_*T*_ is estimated by the predicted target data ${\hat{y}}_{T}$ , using the state matrix for the target dataset **A**_*T*_,

(10) $${\hat{y}}_{T}=A_{T}\left(w_{S}+\delta w\right).$$

Instead of defining the squared error function between the predicted target data ${\hat{y}}_{T}$ and expected target data **y**_*T*_, the L2 regularized version of the squared error function is defined as

(11) $$E_{sq}\left(\delta w\right)={\left({\hat{y}}_{T}-y_{T}\right)}^{2}+\mu \;\delta w^{2},$$

where the parameter *μ* ∈ [0, +∞] is defined as the transfer rate.1Not to be confused with the injection rate *μ*. This transfer rate dictates the amount of information transferred from the training source to the training target domain and needs to be scanned for optimal performance. Minimizing Eq. (11) to *δ***w** results in an expression2Note that the subscript *X*_*T*_ relates here to the training target dataset, while the superscript *X*^*T*^ refers to the transpose of *X*. for the correction weights *δ***w**:

(12) $$\delta w={\left(A_{T}^{T}A_{T}+\mu I\right)}^{-1}\left(A_{T}^{T}y_{T}-A_{T}^{T}A_{T}w_{S}\right),$$

with *I* the identity matrix of size *N* + 1.

The use of transfer learning thus allows one to combine the information gained from two different datasets, via the transfer rate *μ*, so that an optimized performance can be achieved by varying this single parameter.

## 4 Results: transfer learning applied to different tasks

To illustrate the benefits of transfer learning to photonic reservoir computing, we apply this technique to two tasks. For the first task, we simulate data from two Lorenz systems with different parameter values, as is done in [26]. For both Lorenz systems, we sample one of their coordinates to obtain the input samples for the RC system. The data of one of the other spatial coordinates of both Lorenz systems functions as the data samples we want to compute. This allows us to investigate whether transfer learning can reuse information from one Lorenz system to another Lorenz system with slightly different parameter values.

In the second task, we are still working with two Lorenz systems with different parameter values but now we want to quantify the performance of transfer learning when we limit the amount of input data samples for the training target dataset.

### 4.1 Predicting coordinates of Lorenz system

The first task is related to the Lorenz system of ordinary differential equations:

(13) $$\frac{dx}{dt}=\sigma \left(y-x\right)+\zeta_{x}\left(t\right),$$

(14) $$\frac{dy}{dt}=x\left(\rho -z\right)-y+\zeta_{y}\left(t\right),$$

(15) $$\frac{dz}{dt}=xy-\beta z+\zeta_{z}\left(t\right),$$

where we fix the parameters *σ* = 10 and *β* = 8/3. The task we want to solve with our RC system consists of inferring one of the three coordinates, *z*(*t*), from one of the other coordinates, *x*(*t*). We simulate two Lorenz systems, for identical initial conditions (*x*(0) = *y*(0) = *z*(0) = 1) but with different *ρ* parameters and different simulation times. Both Lorenz systems include Gaussian noise for each spatial coordinate, respectively, *ζ*_*x*_(*t*), *ζ*_*y*_(*t*) and *ζ*_*z*_(*t*), with a mean of zero and standard deviation of 0.25. The resulting *x*-, *y*- and *z*-coordinates are normalized to a maximum value of 1.

**Figure 2:**
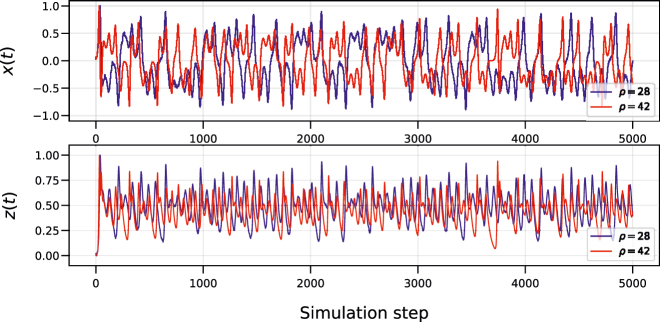
Time traces of the two Lorenz systems with noise, where *σ* = 10, *β* = 8/3 and with different *ρ* (for the *x*-coordinate, top figure, and for the *z*-coordinate, bottom figure).

We integrate the two systems using an Euler scheme with a time step d*t* = 0.01. The training source dataset $D_{S}$ consists of 10^4^ samples of the *x*-coordinate of the Lorenz system where *ρ* = 28 together with the corresponding 10^4^ input samples of the *z*-coordinate. The sampling of the *x*- and *z*-coordinates occurs at the same sampling period as the time steps used during simulation. The samples of the *x*-coordinate are then the input samples of the RC and from the RC output we want to infer the corresponding samples of the *z*-coordinate. Likewise, the training target dataset $D_{T}$ and test dataset consist of 5 × 10^3^ and 2 × 10^3^ data samples of the *x*- and *z*-coordinate of the Lorenz system where *ρ* = 42. After simulation, we discard the first 10 input samples of all three datasets in order to remove the effects of possible transients occurring when switching between datasets with the RC system. Figure 2 shows the time traces for the *x*- and *z*-coordinates of the Lorenz systems with different *ρ* parameters.

These input samples are then injected in an RC system, resulting in three state matrices (for training source dataset dataset, for training target dataset and test dataset). By using different *ρ* parameters for the input data, we are effectively changing the task, while still using the same RC system.

**Figure 3:**
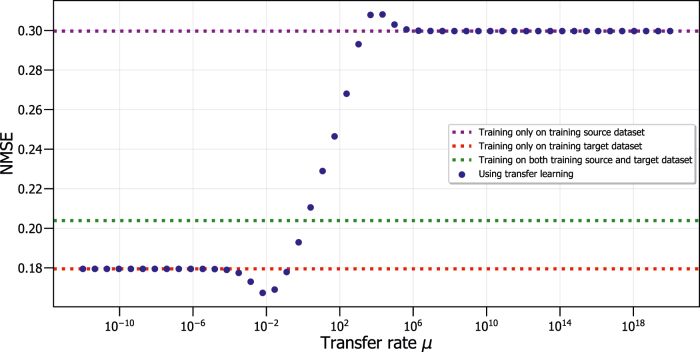
NMSE as a function of the transfer rate *μ* for predicting a Lorenz system with noise with different *ρ* parameter, and comparison to conventional training.

Figure 3 shows the NMSE on the test dataset for *ρ* = 42 as a function of the transfer rate *μ* when we train on various datasets conventionally (without any transfer learning) and with transfer learning. We distinguish three different cases for conventional training, using only the training source dataset $D_{S}$ (shown in purple), using only the training target data $D_{T}$ (shown in red) or using a combined dataset of both the training source dataset and training target dataset (shown in green). In these three cases, we use the weights found during training and apply these during testing, as explained in Section 3.1. All three cases result in horizontal lines in Figure 3, because no transfer learning has been applied and the results therefore do not depend on the transfer rate *μ*. The NMSE values for the three horizontal lines in Figure 3 can be understood conceptually. When we apply conventional training on only the training source dataset, we have a large amount of training samples available, but test on a different Lorenz system (with *ρ* = 42 instead of *ρ* = 28). The resulting NMSE is the highest compared to the other two horizontal lines. When we use conventional training on only the training target dataset, we have the smallest amount of training samples available, but they correspond to the same Lorenz system as the test dataset (both *ρ* = 42), which results in the best NMSE out of the three horizontal lines. If we, however, train on a combined dataset of both training source and training target datasets, we have the largest dataset available of the three cases, and would mainly expect the best performance. This is not the case, because it contains data of a mix of two Lorenz systems with different *ρ* parameters. This results in an NMSE which is situated between the previous two cases.

We can improve the total performance of the RC system by controlling the amount of information transferred from the training source dataset $D_{S}$ to the training dataset $D_{T}$ , by using transfer learning. We show the NMSE in the case where we apply transfer learning, and scan the transfer rate (shown in blue dots) in Figure 3. We observe that when we apply transfer learning, we have an NMSE for *μ* → 0 corresponding to the horizontal line when we only train on training target data. For *μ* → +∞, the NMSE will correspond to the horizontal line where we train conventionally on only the training source dataset. These two extreme regimes of the transfer rate can be explained as follows. For very low transfer rates (*μ* → 0), Eq. (11) will reduce to a least square minimization on the training target dataset. This results in no information transfer from the training source dataset to the training target dataset, thus reducing the training to the conventional RC training on the training target dataset. If the transfer rate becomes very large (*μ* → +∞), the regularizing term of Eq. (11) will become the dominant factor, resulting in *δ***w** becoming very small. This implies that we add no correction to the weights found from the training source dataset, thus transferring no information from the training target dataset to the weights.

For *μ* values between these two extreme cases, we find that around *μ* ≈ 10^−2^ there exist a minimum in the NMSE, after which the NMSE increases drastically to a maximum value. This global NMSE minimum, around *μ* ≈ 10^−2^, indicates that there exists an optimal transfer rate *μ* for which the NMSE is the lowest compared to all other cases, and which thus results in the best performance. At this *μ* value, the information from both the training source and training target dataset is combined optimally to achieve the lowest NMSE. Ultimately, Figure 3 shows that transfer learning results in at least the same NMSE as conventional training, and in general a lower NMSE, for these tasks where we are able to combine information and fine-tune the transfer rate *μ*. The main advantage of transfer learning lies in the fact that when we already have previously trained on a similar dataset (the training source dataset $D_{S}$ ), we are able to use this information and are able to use fewer data samples for a new dataset (the training target dataset $D_{T}$ ). This means that less training would need to be performed and results in a computational speed increase. This will be investigated in Section 4.2.

### 4.2 Influence of training target dataset size

In order to investigate the influence of the size of this training target dataset $D_{T}$ , we again predict the *z*-coordinate of a normalized Lorenz system with noise by the *x*-coordinate, but where we will now change the amount of training target data samples. In order to incorporate transfer learning, we take the state matrix of two different Lorenz systems, one which functions as training source dataset (where *ρ* = 28) and another which functions as training target and test dataset (where *ρ* = 42). For the training source and test dataset, we fix the amount of samples, respectively, to 10^4^ and 2 × 10^3^, while we change and iterate over the amount of training target samples. After simulation, we again discard the first 10 input samples to take into account the transients which can occur when switching between datasets. For every training target size we perform a scan for the value of the transfer rate *μ* which results in the lowest NMSE, as demonstrated in Section 4.1, in order to achieve the most optimal performance per training target dataset size.

**Figure 4:**
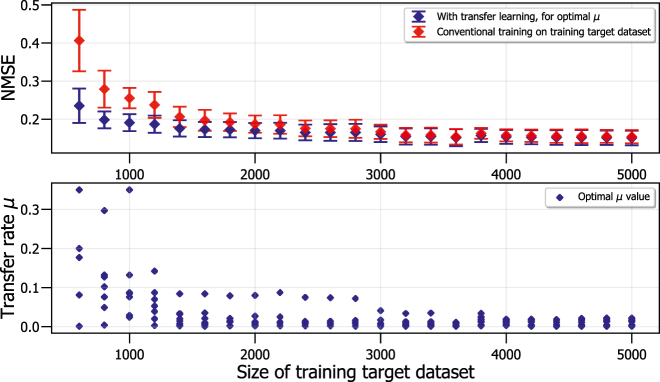
Mean and standard deviation of NMSE as a function of the training target dataset size for predicting the coordinates of Lorenz systems with different *ρ* parameters, using transfer learning or conventional training on the training target dataset (top figure). The most optimal values for the transfer rate *μ* are also shown (bottom figure). In both figures, 7 different masks choices of the RC system are used in the calculations of the mean and standard deviation.

The top panel of Figure 4 shows the NMSE for predicting the *z*-coordinates of a Lorenz system where *ρ* = 42, as training target and test datasets, using information of a Lorenz system where *ρ* = 28, as training source dataset. This is calculated with both the transfer learning technique, where we use the optimal information transfer from training source to training target dataset, and for conventional training, where we only train on the training target dataset and do not use the training source dataset. For every training target dataset size, we have calculated the mean and standard deviation for 7 different iterations, where we have used a different mask *m*(*t*) for every iteration.

We find that the transfer learning method, at optimal *μ*, has a lower NMSE for all training target dataset sizes compared to the conventional training method. This corresponds to the results in Section 4.1. As expected, we observe that for both training techniques the NMSE decreases with increasing amount of training target dataset samples. If the training target dataset becomes small, the NMSE of the conventional training increases drastically, whereas the NMSE for transfer learning remains fairly small. This can be explained by the fact that for the transfer learning case, we already have plenty of information gained from the training source dataset. This is not the case for the conventional training method, where the training target dataset is the only training dataset available. The decreases in NMSE with increasing size of the training target dataset continue to a training target dataset size of around 2 × 10^3^ data samples, from where the NMSE saturates to a quasi constant value.

The bottom panel of Figure 4 shows the values of the most optimal transfer rate *μ* found for each of the 7 iterations per size of the training target dataset. It demonstrates that for increasing size of the training target dataset, the most optimal transfer rate *μ* gradually decreases. This is in agreement with the observations found in Figure 3, where we show that small *μ* values correspond to giving more importance to the training target dataset. This is also the case here, if we have a large training target dataset available. Figure 4 demonstrates that instead of retraining the RC system with a large training target dataset, we can instead reuse already trained weights from another training source dataset combined with transfer learning. For example, we only have to use 1.5 × 10^3^ training target samples combined with already trained weights from a training source dataset, to achieve the same NMSE as conventionally training on a training target dataset of around 2.5 × 10^3^ samples.

## 5 Results: mitigating influence of feedback phase changes on performance of Santa Fe task

Any experimental setup is subject to variations of its internal system parameters, which can be induced by different effects during operation. For example, in our delay-based RC system a change in temperature can lead to a change in the optical length of the delay line. This eventually results in a fluctuating feedback phase, which leads to a worsening of the performance of the reservoir computing system. Typically, the temperature is controlled by thermoelectric cooling (e.g. using Peltier elements). However, very small changes in the feedback phase can still occur, which is why we investigate the use of transfer learning to mitigate performance worsening due to these effects.

Instead of varying the task, which we have done in Sections 4.1 and 4.2, we can instead look into varying the internal RC parameters itself. We thus study if it is possible to compensate for the shift in the feedback phase by applying transfer learning. We try to use transfer learning to quickly calculate the weights, i.e. with a small amount of input samples in the training target dataset used for retraining.

In order to quantify the performance of the RC setups, with varying feedback phase, we choose a one-step ahead prediction task on a time-series used frequently in literature. The used input dataset is the Santa Fe dataset, which consists of 9093 data samples. This dataset is recorded using a far-IR laser in a chaotic regime [27].

In order to investigate the influence of the feedback phase on the performance, we inject – as input data – the first 3010 normalized data samples of the discrete Santa Fe time-series into the RC system. The task is to predict sample *k* + 1 when data up until sample *k* is injected. This results in the state matrix **A** which we use for calculating weights during the training phase. As test dataset, we use the following 1010 data samples of the Santa Fe time-series after the training dataset, with a 10 data sample break, which we also inject into the RC system. After simulation, we discard for both the training and test datasets the first 10 samples to remove any effects of transients occurring from switching datasets. We perform a scan of the feedback phase of the RC system, with *ϕ*_*FB*_ ∈ [0, 2*π*], in order to find the most optimal feedback phase for the RC system. This optimal feedback phase is defined as the feedback phase for which we achieve the best performance, i.e. the lowest NMSE.

**Figure 5:**
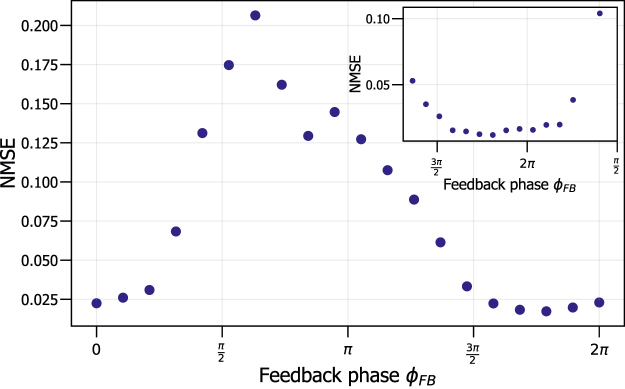
NMSE as a function of the feedback phase of the RC system, for one-step ahead prediction of Santa Fe data, with a zoomed in plot in the top right corner.

Figure 5 shows the NMSE as a function of the feedback phase *ϕ*_*FB*_ for the one-step ahead prediction of the Santa Fe time-series. In the top right a zoom is shown of the plot near its minimum NMSE. Simulations of RC systems with *ϕ*_*FB*_ = 0 typically result in NMSE values around 0.01 and 0.02 for the Santa Fe one-step ahead predictions, which agrees with the values found in Figure 5 [14, 24].

In Figure 5, we find that the performance of our RC system on this task is very sensitive to the feedback phase *ϕ*_*FB*_. Feedback phases between *ϕ*_*FB*_ ∈ [3*π*/2, 2*π*] result in the best NMSE, while feedback phases outside this range perform rather poorly, with higher NMSE. We find that a value for the feedback phase around *ϕ*_*FB*_ ≈ 5.68 corresponds to the lowest NMSE. Therefore, we use this feedback phase as the most optimal feedback phase for our RC systems.

Having found the most optimal feedback phase, we again inject the first 3010 data samples of the normalized Santa Fe time-series into our RC system, which has a constant feedback phase *ϕ*_*FB*,*S*_ = 5.68. The resulting state matrix **A**_*S*_ corresponds to the training source response $D_{S}$ . In order to apply transfer learning, we need to define a training target response $D_{T}$ . This training target response is defined as the state matrix **A**_*T*_ resulting from injecting the first 510 data samples of the normalized Santa Fe time-series into our RC system, but with a different feedback phase *ϕ*_*FB*_ (where *ϕ*_*FB*,*T*_ ∈ [5.70, 5.71, 5.72, 5.73]). The values for these feedback phases are small deviations from the most optimal feedback phase, with the maximum deviation only being 0.5%. In a similar fashion, we define the test response as the next 1010 data samples, with a 10 sample break, after the training source data samples of the same time-series, for the same feedback phase *ϕ*_*FB*_ as for the training target response. After simulation, we again discard the first 10 samples of the responses to remove any effects of transients occurring from switching responses.

**Figure 6:**
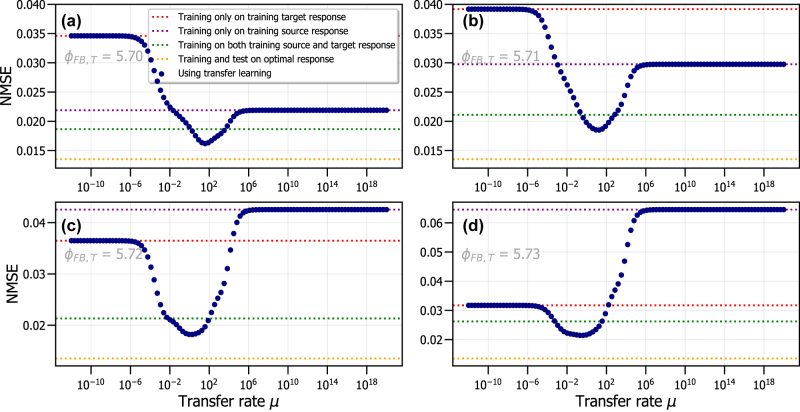
NMSE as a function of the transfer rate *μ*, using a training source response with *ϕ*_*FB*,*S*_ = 5.68 and four training target responses originating from different RC systems: *ϕ*_*FB*,*T*_ = 5.70 (a), *ϕ*_*FB*,*T*_ = 5.71 (b), *ϕ*_*FB*,*T*_ = 5.72 (c) and *ϕ*_*FB*,*T*_ = 5.73 (d).

Figure 6 shows the NMSE corresponding to the different training schemes. It shows the performance when we conventionally train on only the training target response (in red), on only the training source response (in purple), on a combined response of both training source and training target data (in green) and when training on the optimal RC system (with the same optimal feedback phase *ϕ*_*FB*,*S*_ = 5.68 for the training response as the test response). This last NMSE is used as the reference NMSE value, because it corresponds to the best NMSE value we can expect, since the *ϕ*_*FB*_ of the RC system is optimal and identical for both the training and test response. All of the previously mentioned NMSE values do not vary with the transfer rate *μ*, since no transfer learning has been used. The training scheme where we apply transfer learning between the training source and training target response (shown with blue dots) varies with the transfer rate *μ*.

These results are shown in Figure 6 for the four investigated feedback phases *ϕ*_*FB*,*T*_ of the training target response. The feedback phases closest to the optimal *ϕ*_*FB*,*S*_ have a better NMSE when performing conventional training on only the training source response compared to conventional training on only the training target response. This is the case for Figure 6(a) and (b), where the feedback phase of the training source system is similar to that of the training target system. However, when the feedback phase of the training target response *ϕ*_*FB*,*T*_ is too different from that of the training source response *ϕ*_*FB*,*S*_, training on the training source response results in a large NMSE, surpassing that of the situation when the RC is trained on the training target response. This result can be seen in Figure 6(c) and (d). This again shows that the value of the feedback phase for the training target response is very sensitive for the performance of the RC, which was already indicated by Figure 5.

The performance of conventional training on a combined response consisting of both the training source and training target response is also shown in Figure 6 for all four cases. This combined response can therefore be seen as a response for which the feedback phase has changed during the experiment. It contains the most amount of data samples, in total 3500 samples. Training conventionally on this combined response always results in better performance when compared to purely training conventionally on either the training source or training target response. However, we are able to improve this performance by introducing transfer learning, which controls the information transfer between both training source and training target responses.

We observe in Figure 6 that, similar to Figure 3, for small *μ* we have the situation corresponding to conventionally training only on the training target response. When *μ* is further increased, the NMSE will decrease until an optimal *μ* value is found. At this optimal transfer rate, around *μ* ≈ 10^2^ to *μ* ≈ 1, all four figures of Figure 6 show a minimum value for the NMSE. This point corresponds to the most optimal transfer rate of information between training source and training target response and thus results in the best performance using both responses. For large *μ*, the situation is similar to training on only the training source response. This is to be expected, and is also described in Section 4.1.

From Figure 6, we observe that if we start from the optimal feedback phase *ϕ*_*FB*,*S*_, and there is a small drift in the feedback phase, that retraining using transfer learning is the best option. This is due to a better performance than conventional training on only the training target response and also because it is more time and energy efficient than conventional training on the combined response of the training target and training source response. We also observe that if *ϕ*_*FB*,*T*_ is strongly different from *ϕ*_*FB*,*S*_, we do not get good performance from transfer learning. This is in agreement with the results of Figure 5, where we have shown that achieving a good performance for these phases is simply not possible.

We have investigated the effect of phase variations in the feedback term and found that the performance quickly deteriorates when this parameter is changed, even with small variations. We found that transfer learning is slightly able to limit this worsening in performance, but only within a limited percentage from the optimal value for the feedback phase. We thus conclude that transfer learning can only be used when confronted with small changes in the feedback phase of delay-based photonic RC systems. In the next section, we investigate the performance when the RC system is retrained using transfer learning when three other parameters are varied. These parameters have less influence on the performance of the RC systems, compared to the feedback phase, and are investigated for larger parameter variations.

## 6 Results: mitigating influence of multiple parameter variations on performance of Santa Fe task

In this section, we investigate the performance of RC systems when they are retrained using transfer learning, and when three parameters are varied: the injection rate *μ*, the feedback rate *η* and the excess pump current rate Δ*I*/*e*.

The injection of Santa Fe data is identical to the procedure described in Section 5, with the only difference that instead of varying the feedback phase, we vary the injection rate, the feedback rate and the excess pump current rate. The optimal values for these three parameters are defined in Table 1 (denoted here as *μ*_opt_, *η*_opt_, Δ*I*_opt_/*e*) and are used for creating the state matrix **A**_*S*_. We again define the training target response $D_{T}$ as the state matrix **A**_*T*_ resulting from injecting normalized Santa Fe samples into our RC system, but with different values for *μ*, *η* and Δ*I*/*e*. The values for these three parameters are defined as *xμ*_opt_, *yη*_opt_, *z*Δ*I*_opt_/*e* where (*x*, *y*, *z*) are three random numbers drawn from a Gaussian distribution with a mean of 1 and standard deviation given by *σ*. This *σ* dictates the amount of deviation from the optimal values of *μ*_opt_, *η*_opt_, Δ*I*_opt_/*e*, and is varied over 20 different values ranging between 0.01 and 0.20. For every *σ*, we repeat the simulation 10 times, and thus with 10 different (*xμ*_opt_, *yη*_opt_, *z*Δ*I*_opt_/*e*) combinations. This implies that for each *σ*, we repeat the experiment multiple times, and are thus able to achieve a better statistical result. As defined in Section 5, the test response is also created using the same parameters as for the training target response.

**Figure 7:**
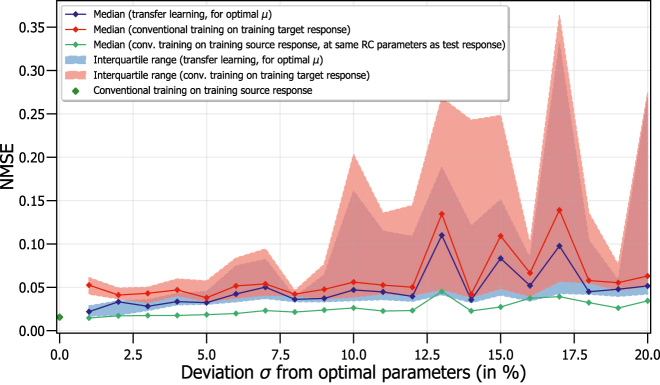
Median and interquartile range of NMSE as a function of the deviation from the optimal parameters of injection rate, feedback rate and excess pump current rate, for one-step ahead prediction of Santa Fe data. 10 different parameter combinations are used for the calculations of the median and interquartile range, where the same mask is reused for every realization.

For each of the 10 iterations, we perform a scan for the value of the transfer rate which results in the lowest NMSE. This is done in order to achieve the most optimal performance for every iteration. Figure 7 shows the NMSE for the one-step ahead prediction of the Santa Fe dataset. In this figure, we show the median and interquartile range, calculated over the 10 different parameter combinations, each with identical mask. This is done for the case when we use transfer learning with the training source and target response, at the most optimal transfer rate (in blue), and when we conventionally train on the training target response (in red). As a reference value, we also show the performance without any deviations (i.e. *σ* = 0) in the RC’s parameters where we conventionally train on the training source response, at the optimal values for the three parameters (in dark green). Additionally, we show the results when we inject the entire source input samples into the RC system with the same parameter combination as the test response, and conventional train on this dataset. These results are shown for various *σ* (in light green) and represent the best possible results. We use the median and interquartile range, instead of the mean and standard deviation, to show the spread of the NMSE since they are less influenced by large outliers of the NMSE.

Figure 7 shows that the median NMSE is consistently lower when using transfer learning compared to conventional training on the training target response, as shown by the blue line always being below the red line. The median when using transfer learning remains around NMSE ≈0.03 for *σ* ⪅ 5%, whereas for conventional training on the target response we typically obtain NMSE ≈0.05 . This should be compared with the best possible result of the NMSE being around ≈0.02 for conventional training. This is referred to as the best result for conventional training, as it uses 3000 data samples in an RC system which has the same parameter combination as the test set for its training source response, as opposed to only 500 samples for the training target response. This means that these best possible results correspond to fully retraining the system, which is very time-intensive and we thus want to avoid, while with transfer learning we only partially retrain the system.

In Figure 7, we observe that the median NMSE for transfer learning and conventional training increase when *σ* increases. The increase of the median NMSE with *σ* is, however, not monotone in both cases due to the fact that only 10 random iterations are used for the calculation of the median. Therefore, it is possible that certain parameter combinations were chosen, even at increased *σ*, where a good performance, and thus low NMSE, is found (e.g. around *σ* = 8%). However, we observe that for transfer learning, the increase or decrease in median NMSE with *σ* is also present for the conventional learning case. This can be explained by the fact that the medians are calculated with the same parameter combinations of *μ*, *η* and Δ*I*/*e*. This implies that a well-performing parameter combination will lead to a good NMSE, for both the transfer learning case and conventional learning case.

Additionally, the interquartile range is also smaller, with slightly lower NMSE, when using transfer learning, indicating that transfer learning is able to improve the performance of RC systems. However, for both cases, the fluctuations for the interquartile range increase with increasing parameter deviation. This can be explained by the fact that for increasing *σ*, the probability of having parameter combinations which result in a poor performance increases, due to the larger parameter deviations. Due to these fluctuations, we limit the applicability of transfer learning to around parameter deviations of *σ* ≈ 10%, as the NMSE becomes too large for higher *σ*.

Finally, we conclude that transfer learning can be used for parameter deviations of *μ*, *η* and Δ*I*/*e* up to *σ* ≈ 10%, where a lower NMSE is found when compared to conventional training on the training target response, and where the fluctuations in NMSE remain small.

## 7 Conclusions

In this work, we have numerically investigated the application of a novel training scheme, transfer learning, for delay-based reservoir computing with semiconductor lasers. With transfer learning, one is able to control the information transfer between two training sets, a training source and training target dataset, by controlling the transfer rate parameter, *μ*. This allows one to combine previous information and reuse previously found weights, resulting in less training data being required in the training target dataset. We have found that by using transfer learning, we are able to increase the performance on predicting coordinates of a Lorenz system with different parameters, even with relatively small training data available on that target Lorenz system. We have also investigated how small this training target dataset can be made and still result in improved performance compared to conventionally training on a training target dataset, when predicting the behaviour of a Lorenz system. We have found that using transfer learning with only 1.5 × 10^3^ training target samples, combined with 10^4^ training source samples, have the same performance as conventionally training on 2.5 × 10^3^ samples as training target dataset. Since we do not have to retrain the weights corresponding to the training source dataset, this implies that ultimately we have to perform less retraining when the weights corresponding to the training source dataset are already available. Finally, we have also investigated the possibility of using transfer learning to compensate for the worsening of reservoir computing performance by parameter variations. In order to study this, we have first looked into the effect of changes in the feedback phase of the reservoir computing systems. We are able to update the weights – originally obtained at the optimum feedback phase *ϕ*_*FB*_ – when the feedback phase drifts by using a limited amount of training target samples combined with transfer learning. By training on a reservoir computing system at the most optimal feedback phase, we were able to mitigate, to a certain degree, this performance worsening for slightly varying feedback changes. If we, however, use transfer learning when confronted with parameter deviations of the injection rate, feedback rate and excess pump current rate, we were able to achieve better results, up to large parameter deviations. Therefore, we conjecture that transfer learning can be used to enhance the performance of other photonic RC systems, which are also suffering from internal parameter drift.
